# Evaluation of *Candida* Colonization and Specific Humoral Responses against *Candida albicans* in Patients with Atopic Dermatitis

**DOI:** 10.1155/2015/849206

**Published:** 2015-04-06

**Authors:** Ghaffari Javad, Mehdi Taheri Sarvtin, Mohammad Taghi Hedayati, Zohreh Hajheydari, Jamshid Yazdani, Tahereh Shokohi

**Affiliations:** ^1^Department of Pediatrics, School of Medicine, Mazandaran University of Medical Sciences, Sari, Iran; ^2^Department of Medical Mycology and Parasitology, School of Medicine, Jiroft University of Medical Sciences, Jiroft, Iran; ^3^Department of Medical Mycology and Parasitology, School of Medicine, Mazandaran University of Medical Sciences, Sari, Iran; ^4^Invasive Fungi Research Center, Mazandaran University of Medical Sciences, Sari, Iran; ^5^Department of Dermatology, School of Medicine, Mazandaran University of Medical Sciences, Sari, Iran; ^6^Department of Statistics, School of Health, Mazandaran University of Medical Science, Sari, Iran

## Abstract

The aim of this study was to assess the candidal colonization and specific humoral responses against *Candida albicans* in patients with atopic dermatitis. One hundred patients with atopic dermatitis and 50 healthy individuals were enrolled in the study. Skin and oral specimens from all participants were cultured on CHROMagar *Candida* medium. Isolated yeasts were identified by using the sequence of the D1/D2 domain of the 26S rRNA gene. ELISA was used for detection of IgM, IgA, and IgG antibodies against *C. albicans* in sera of participants. *Candida* species were isolated from the skin and oral cavity of 31% of the patients and 12% of the controls. There was no significant difference between *Candida* colonization in patients and controls (*P>0.05*). *Candida albicans* was isolated from the skin and oral cavity of 23% of the patients and 6% of the controls (*P< 0.05*). There were no significant differences between serum levels of IgM and IgA in patients and controls (*P>0.05*). Serum level of IgG was significantly lower in patients than in controls (*P<0.05*). Type of *Candida* colonization can change in patients with atopic dermatitis. In addition, these patients have abnormalities in the production of antibodies against *Candida albicans* that may have a role in the pathogenesis of atopic dermatitis.

## 1. Introduction

Atopic dermatitis (AD) is an inflammatory, relapsing, itchy, and noncontagious skin disorder that is associated with asthma and hay fever [1]. This disease is the most common skin disorder in children 7 years old and almost 18% of children have had or have a history of atopic dermatitis [2]. The combination of several factors such as genetic predisposition, skin barrier defects, immunological factors, and environmental factors such as food, house dust mites, and specially microorganisms including* Candida* spp.,* Malassezia* spp., and* Staphylococcus aureus* might contribute to the onset and exacerbation of this disease [2].* Candida* species is one of the most important fungal colonizers on the skin and mucosal surfaces of the body such as genitourinary tract, oral cavity, and gastrointestinal tract [3, 4].* Candida* can cause a wide range of disorders such as vulvovaginitis, oral thrush, and skin and diaper rash as well as life threatening diseases in immunocompromised patients [3–6]. So far, over 200 species of* Candida* have been identified, but among them* C. albicans*,* C. glabrata, C. tropicalis,* and* C. parapsilosis* are responsible for the majority of candidal infections [3–5].* Candida* species are able to stimulate the immune system causing or worsening clinical conditions of atopic dermatitis via secretion of variety of allergens and antigens [2]. Therefore the colonization of* Candida* species in patients with this disease should be assessed and controlled. So far only two studies investigated* Candida* colonization in skin and oral cavity of patients with atopic dermatitis and have provided different results [7, 8].* Candida* species can play an important role in the pathogenesis of atopic dermatitis via stimulation of humoral immune system and reaction with immunoglobulins [2]. Some researchers believe that production of specific antibodies against* Candida albicans* is associated with increased severity of atopic dermatitis. But all the studies only examined the production of IgE antibody against* Candida albicans* in these patients [2, 9]. Therefore this study was designed to investigate the colonization of* Candida* species on the skin and oral cavity and production of IgM, IgG, and IgA antibodies against* Candida albicans* in patients with atopic dermatitis.

## 2. Materials and Methods

### 2.1. Patients

One hundred patients with atopic dermatitis and 50 healthy individuals as control group from January 2011 to March 2012 were enrolled in the study. The patients and controls filled out the consent form to participate in research and the study was approved by the ethical committee of Mazandaran University of Medical Sciences, Sari, Iran.

Control subjects were selected from persons who were referred for cosmetic problems. People who had diabetes and those who had used broad spectrum antibiotics and steroids as well as pregnant patients were excluded from the study. In order to assess clinical severity of the disease, the SCORAD (SCORing Atopic Dermatitis) index was calculated as elucidated by Kunz et al. in 1997 [10]. Based on this definition, clinical severity of atopic dermatitis was categorized to mild (SCORAD index < 10), moderate (SCORAD 10–18), and severe (SCORAD > 18).

### 2.2. Mycological Investigation

The samples were collected from oral cavity and skin by swab and scalpel, respectively. All of the samples were cultured on CHOROMagar* Candida* medium (CHOROMagar Company, Paris, France). The isolated species of* Candida* were subcultured on Sabouraud's dextrose agar containing chloramphenicol (SC) and incubated at 27°C for 4 days.

### 2.3. Molecular Investigation

The DNA of the isolated yeasts was extracted according to the procedure of Yamada et al. [11].

Yeasts were identified to the species level using sequence analysis of the D1/D2 domain of the 26S ribosomal RNA gene. For amplification of the D1/D2 domain, the external primers NL-1 (5′-GCA TAT CAA TAA GCG GAG GAA AAG-3′) and NL-4 (5′-GGT CCG TGT TTC AAG ACG G-3′) [12] were used. The reactions were performed in an automatic thermal cycler (C1000 Thermal Cycler, Bio-Rad Laboratories) with initial denaturation at 95°C for 3 min; 35 cycles at 95°C for 30 s, 56°C for 30 s, and 72°C for 30 s; final extension at 72°C for 7 min. The quality of PCR products was determined by electrophoresis in 1% (w/v) agarose gel in 1x TBE (89 mM Tris-base, 89 mM boric acid, and 2 mM EDTA) at 80 V for 2 h. GenRuler DNA ladder mix was used as a marker. Sequencing of the purified PCR products was performed using the primers NL-1. Sequences were aligned to the 26S rRNA gene sequences obtained from the National Center for Biotechnology Information (NBCI) Genbank database (http://www.ncbi.nlm.nih.gov/) and yeast species were identified by searching databases using the BLAST algorithm.

### 2.4. Detection of Anti-*C. albicans* Antibodies

Serum IgG, IgM, and IgA levels were measured with enzyme-linked immunosorbent assay (ELISA) test kits (Genesis Diagnostic, England) according to the manufacturer's instructions.

### 2.5. Statistical Analysis

Numbers of individuals with yeast growth were compared with the Chi-Square test. Independent *t*-test was used to compare the results of IgG and IgM assay in two groups. *P* values less than 0.05 were considered significant. The results of IgA assay were analyzed by Kruskal-Wallis test and paired comparisons were performed by means of the Mann-Whitney *U* test. *P* values less than 0.015 were considered significant. The correlation between* Candida* carriage and levels of antibodies against* C. albicans* with the severity of disease (SCORAD index) were examined by means of Chi-Square test and Pearson's_correlation_test, respectively. Logistic regression was used to control confounding effects of age and sex.

## 3. Results

**Figure 1:**
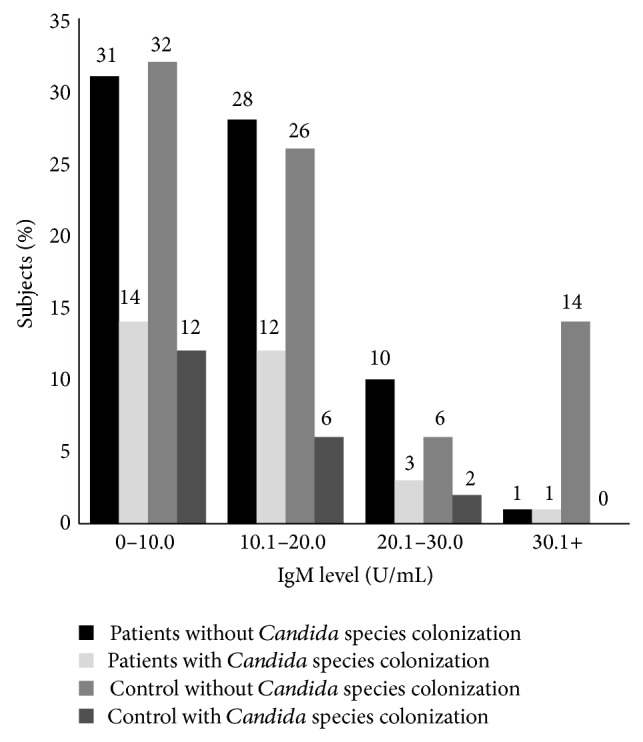
Distribution of serum level of IgM specific against* Candida albicans* in patients with atopic dermatitis and control (mean of IgM in patients: 11.6 ± 7.7 and mean of IgM in controls: 14.68 ± 12.00).

**Figure 2:**
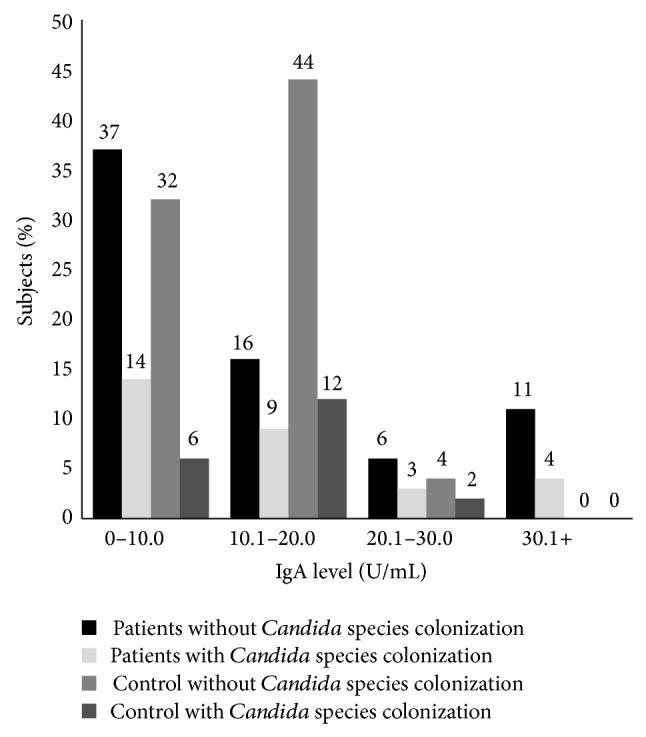
Distribution of serum level of IgA specific against* Candida albicans* in patients with atopic dermatitis and control (mean of IgA in patients: 13.18 ± 12.83 and mean of IgA in controls: 13.8 ± 4.74).

**Figure 3:**
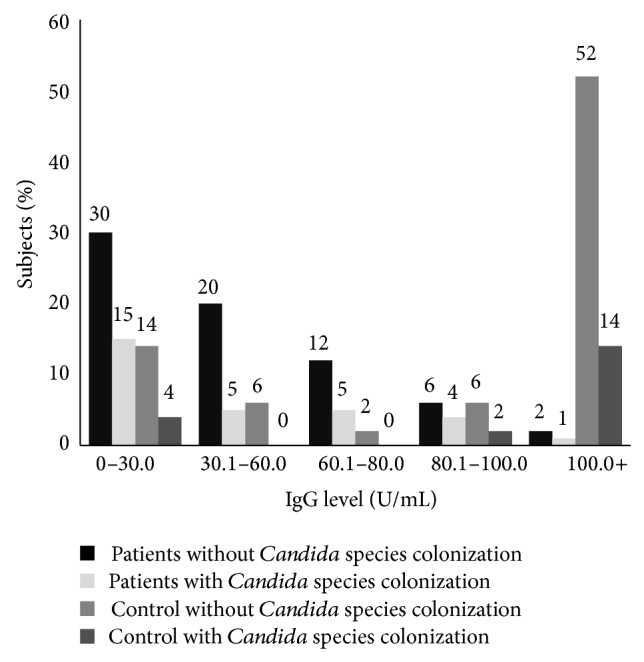
Distribution of serum level of IgG specific against* Candida albicans* in patients with atopic dermatitis and control (mean of IgG in patients: 42.64 ± 31.5 and mean of IgG in controls: 90.03 ± 40.21).

In this study, 100 patients (27 male and 73 female; age mean 12.1 ± 11.5 years) and 50 controls (22 male and 28 female; age mean 39.9 ± 11.45 years) were examined. By logistic regression analysis, age and sex do not influence* Candida* colonization and levels of antibodies (*P* > 0.05). Thirty-one percent of patients and 24% of controls were colonized by yeast species (*P* = 0.243).* Candida* species were isolated from the oral cavity of 30 (30%) patients and 10 (20%) controls (*P* = 0.133).* Candida* species were isolated from the skin of 2 (2%) patients and 2 (4%) of controls. Twenty-nine percent of patients and 22% of controls were colonized by only one yeast species. Two percent of patients and 2% of controls were colonized by two different yeast species.* Candida albicans* and* Candida glabrata* were the most common yeast species isolated from patients and control, respectively. Isolated yeast species are listed in Table 1. Twenty-three percent of patients and 6% of controls were colonized by* Candida albicans* (*P* = 0.006). SCORAD index < 10, SCORAD index 10–18, and SCORAD index > 18 were observed in 20%, 70%, and 10% of the patients with atopic dermatitis, respectively. 20% of patients with mild disease, 17.5% of patients with moderate disease, and 10% of patients with severe disease were colonized by* Candida* species. IgG and IgM titers against* Candida* show normal distribution in patients and control group. The mean level of IgM against* C. albicans* in patients and controls was 11.6 U/mL and 14.68 U/mL, respectively (*P* = 0.112). The mean level of IgG against* C. albicans* in patients and controls was 42.64 and 90.01 U/mL, respectively (*P* < 0.001). IgA levels against* Candida* show abnormal distribution in patients and control group. The mean level of IgA against* C. albicans* in patients and controls was 13.18 U/mL and 13.8 U/mL, respectively (*P* = 0.022). The Pearson correlation between levels of IgM, IgG, and IgA and severity of disease was 0.049, 0.071, and −0.022, respectively (*P* > 0.05). Fourteen percent of patients and 12% of controls with* Candida* colonization showed IgM level less than 10 U/mL (Figure 1). Fourteen percent of patients and 6% of controls with* Candida* colonization had IgA level less than 10 U/mL (Figure 2). The IgG levels of 15% of patients and 4% of controls with* Candida* colonization were less than 30 U/mL (Figure 3).

## 4. Discussion

In the present study, there was no significant difference between the rate of* Candida* species colonization in the oral cavity and skin of patients with atopic dermatitis and controls. This result is incompatible with Henseler and Tausch's study [8] and is compatible with Leibovici et al.'s study [7]. However, the differences in the results of these studies may be due to differences in race, age, and disease severity of the study population. In the present study although there was no significant difference between the colonization of* Candida* species in the patients and controls, impaired immune systems of patients with atopic dermatitis can react to the normal rate of* Candida* species colonization and alter the course of the disease [2]. In the present study,* Candida albicans* colonization in the oral cavity of patients was significantly higher than controls.* Candida albicans* is the most common and important species [13]. Protein compounds of this species such as proteins 27, 37, 43, 46, 125, and 175 kDa can play an important role in the pathogenesis of atopic dermatitis via stimulation of immune system and reaction with immunoglobulins [13]. Some researchers have reported that increased severity of atopic dermatitis is associated with the production of* Candida albicans*-specific antibodies [2, 9, 14]. In Matsumura et al.'s study [14], level of IgE antibody against* Candida albicans* in patients with atopic dermatitis was significantly higher than in controls. In Faergemann study, pediatric patients and patients with severe atopic dermatitis had higher level of IgE antibody against* Candida albicans* than those with mild disease and controls [2]. In Matsumura et al.'s study [14], 85% of patients with atopic dermatitis have a high level of IgE antibody against* Candida albicans*. As noted above, most studies examined specific IgE to* Candida albicans* and its immunoglobulin-reactive protein in patients with atopic dermatitis. Hence, in this study, the production of other classes of antibody to* Candida albicans* including IgG, IgA, and IgM was evaluated. In the present study, there were no significant differences between the levels of IgM and IgA antibodies against* Candida albicans* in serum of the patient and control groups, but level of specific IgG to* Candida albicans* was significantly lower in patients than in controls. IgG is the major immunoglobulin in normal human serum and is a key player in the humoral immune response [6]. The reduction in specific IgG production may be the etiology for the increased* Candida albicans* colonization in patients with atopic dermatitis. According to the survey which was conducted, so far the levels of specific antibodies against* Candida albicans* have not been evaluated in serum of patients with atopic dermatitis. So it seems that the present study is the only study that has addressed this issue. In our study, there were no significant differences among antibody levels, severity of illness, and intensity of* Candida* colonization. In present study, we applied logistic regression to control confounding effects of age and sex. Therefore age and gender had no effect on the results of this study.

The results of this study showed that type of* Candida* colonization can change in patients with atopic dermatitis. Moreover, these patients have abnormalities in the production of antibodies against* Candida albicans* that may have a role in the pathogenesis of atopic dermatitis.

## Figures and Tables

**Table 1:** Yeast species isolated from patients with atopic dermatitis and controls.

Yeast species	Oral specimens		Skin specimens	
	Patients *N* (%)	Controls *N* (%)	Patients *N* (%)	Controls *N* (%)
*Candida albicans *	23 (74.2%)	3 (23%)	0 (0%)	2 (100%)
*Candida glabrata *	0 (0%)	4 (30.9%)	0 (100%)	0 (0%)
*Debaryomyces hansenii *	2 (6.45%)	0 (0%)	2 (0%)	0 (0%)
*Candida tropicalis *	0 (0%)	1 (7.7%)	0 (0%)	0 (0%)
*Candida dubliniensis *	2 (6.45%)	0 (0%)	0 (0%)	0 (0%)
*Candida parapsilosis *	2 (6.45%)	3 (23%)	0 (0%)	0 (0%)
*Issatchenkia orientalis *	2 (6.45%)	2 (15.4%)	0 (0%)	0 (0%)

Total	31 (100%)	13 (100%)	2 (100%)	2 (100%)
